# Immunohistochemistry for p16 and p53 Provides Substantial Agreement With Molecular Tests in Penile Cancer

**DOI:** 10.1111/pin.70138

**Published:** 2026-06-16

**Authors:** Jan Hrudka, Jan Hojný, Eva Krkavcová, Jiří Dvořák, Zuzana Prouzová, Michaela Kendall Bártů, Nicolette Zavillová, David Čapka, Radoslav Matěj, Petr Waldauf

**Affiliations:** ^1^ Department of Pathology, 3^rd^ Faculty of Medicine Charles University, University Hospital Kralovske Vinohrady Prague Czech Republic; ^2^ Department of Pathology, 1^st^ Faculty of Medicine Charles University, General University Hospital Prague Czech Republic; ^3^ Department of Urology, 3^rd^ Faculty of Medicine Charles University, Thomayer University Hospital Prague Czech Republic; ^4^ Department of Urology, 3^rd^ Faculty of Medicine Charles University, University Hospital Kralovske Vinohrady Prague Czech Republic; ^5^ Department of Pathology and Molecular Medicine, 3^rd^ Faculty of Medicine Charles University, Thomayer University Hospital Prague Czech Republic; ^6^ Department of Anaesthesia and Intensive Care Medicine, 3^rd^ Faculty of Medicine Charles University, University Hospital Kralovske Vinohrady Prague Czech Republic

**Keywords:** Human papillomavirus (HPV), Immunohistochemistry, Next generation sequencing (NGS), Penile squamous cell carcinoma (pSCC), Prognostic biomarkers, quantitative PCR (qPCR), TP53 mutation

## Abstract

Penile squamous cell carcinoma (pSCC) is a rare cancer associated with a relatively poor prognosis. The WHO classifies pSCC into HPV‐associated and HPV‐independent subtypes, with HPV‐positive pSCCs showing slightly better outcomes. A key negative prognostic marker is *TP53* mutation. The aim of this study was to evaluate the concordance of (1) p16 immunohistochemistry with HPV status determined by PCR, (2) p53 immunohistochemistry with *TP53* mutational status obtained by NGS, and (3) prognostic impact of overexpression and null phenotype p53 immunohistochemistry profiles. Analyzing invasive pSCC from 209 patients, we assessed concordance between p16 immunohistochemistry and HPV status (*n* = 132) determined by quantitative PCR, obtaining Cohen's kappa Κ = 0.62 (*p* < 0.0001). For p53 immunohistochemistry versus *TP53* status by NGS (*n* = 145), Cohen's kappa reached Κ = 0.693 (*p* < 0.0001). We found worse overall survival (Hazard ratio = 3.33, 95% CIs 1.92–5.56, *p* < 0.0001) in p53 mutated cases (*n* = 38, both overexpression and null phenotype) compared to wild type (*n* = 171), but without a significant difference between both mutated profiles. Both HPV status and p53 profile can be reliably assessed by immunohistochemistry with substantial agreement. Patients with a mutated p53 profile showed significantly worse survival, irrespective of the mutated profile variant.

## Graphical abstract

In penile squamous cell carcinoma, p16 and p53 immunohistochemistry showed substantial concordance with molecular testing (Cohen's kappa 0.6–0.7). Aberrant p53 expression profiles (overexpression or null phenotype) were associated with significantly worse survival. These findings support incorporating p53 immunohistochemistry into routine prognostic assessment.

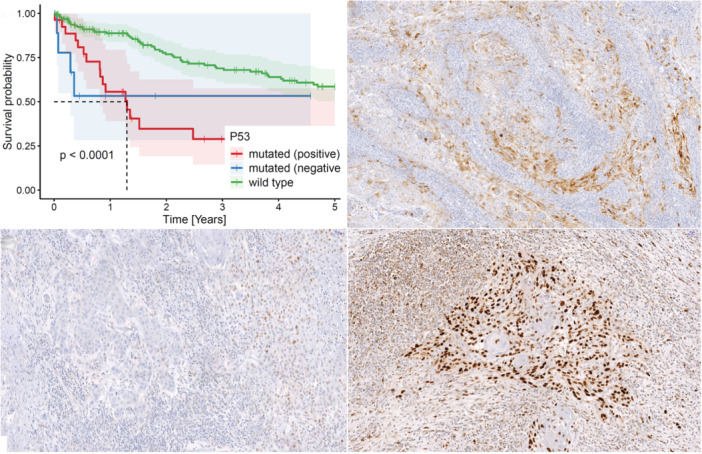

## 1 Introduction

Penile squamous cell carcinoma (pSCC) accounts for about 0.2% of all cancer incidences and 0.1% of cancer‐related mortality [1]. Although rare, pSCC has the poorest prognosis among male genitourinary malignancies [2]. In the 2020s, a global effort emerged to advance prognostic models through the use of larger patient cohorts.

The WHO classification distinguishes pSCCs based on their association with human papillomavirus (HPV), categorizing them as either HPV‐associated or HPV‐independent. Both types are approximately equally frequent. HPV‐associated tumors exhibit specific patterns of oncogenic genomic alterations [3, 4]. Several methods are available to determine HPV status in pSCC, including p16INK4a (p16) immunohistochemistry, polymerase chain reaction (PCR), and next‐generation sequencing (NGS). p16 immunohistochemistry is the most widely used diagnostic tool in routine practice. Block‐type positivity, defined as diffuse cytoplasmic and nuclear staining in ≥ 70% of tumor cells, is considered a surrogate marker for HPV‐driven oncogenesis [5]. Recently, a meta‐analysis documenting better prognosis in HPV‐associated pSCC was published [6, 7].

There is increasing evidence of the negative prognostic impact of *TP53* alterations, present in 10%–48% of pSCCs [4, 8]. Independently from p16/HPV status, *TP53* alterations lead to disrupted cell cycle, decreased apoptosis, epithelial‐to‐mesenchymal transition and invasion, underlying their aggressive phenotype [9]. Previously, in a relatively large cohort, we documented a strong association of p53 mutated status with tumor budding and poor prognosis in pSCC [10, 11]. The expression profile of p53 can be detected immunohistochemically. Physiological tissues and tumors without *TP53* alterations exhibit heterogeneous nuclear staining, referred to as the wild‐type pattern. In cells harboring *TP53* missense mutations, dysfunctional undegradable p53 protein accumulates within the nucleus, resulting in a uniform and strong uniform nuclear immunohistochemical signal in ≥ 80% tumor cells (overexpression) [12]. In the case of nonsense mutations or deletions of *TP53*, the null phenotype is observed, in which the nuclei of tumor cells are completely negative.

The aim of this study was to evaluate the concordance of (1) p16 immunohistochemistry with HPV status determined by PCR, (2) p53 immunohistochemistry with *TP53* mutational status obtained by NGS, and (3) prognostic impact of different (overexpression vs. null phenotype) p53 immunohistochemistry profiles in a large cohort of pSCC.

## 2 Methods

We utilized our previously published data comprising 209 surgically treated or biopsied and histologically verified pSCC with formalin‐fixed paraffin‐embedded (FFPE) tissue and known follow‐up data available [11]. In 145 cases, we examined p53 expression by immunohistochemistry and by an NGS panel comprising 355 tumor‐related genes. In 132 cases, we examined p16 expression by immunohistochemistry and by quantitative PCR using the Anyplex II HPV28 Detection qPCR kit (Seegene), as described in detail elsewhere [4, 10]. The study was approved by the institutional review board and the institution's ethics committee, approval number EK‐VP1261012020. The informed consent was waived due to the retrospective way of the study. For the agreement rate assessment in both p53 and p16/HPV, we used Cohen's kappa calculation. For overall survival (OS) analysis, Kaplan–Meier analysis was performed using the log‐rank test and confidence intervals calculated by the log‐log method, followed by restricted mean survival time (RMST) analysis. OS analysis was performed based only on immunohistochemical p53 profiles. To calculate the hazard ratio (HR) for each parameter, univariate Cox regressions with 95% CIs were performed. All statistical analyses were performed using R software, version 4.5.1.

## 3 Results

**Figure 1:**
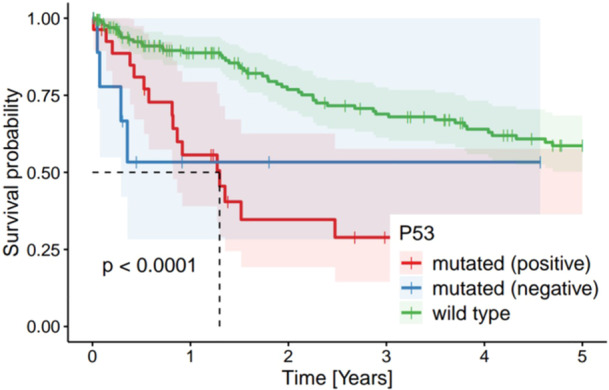
Kaplan Meier curves showing significantly shorter overall survival in patients with p53 mutated immunoprofile in penile squamous cell carcinoma, with no apparent difference between p53 overexpression (positive) and null phenotype (negative).

Cohen's kappa results are summarized in Table 1, the OS analysis in Figure 1. The raw data on Cohen's kappa analysis are in Supplementary 1, on the OS analysis in Supplementary 2. For p53 immunohistochemistry/*TP53* by NGS, Cohen's kappa reached Κ = 0.693 (*p* < 0.0001). For p16/HPV qPCR, Cohen's kappa reached Κ = 0.62 (*p* < 0.0001). Both HPV status and p53 profile can be diagnosed by immunohistochemistry with substantial agreement. The cases with discordant immunohistochemistry/molecular findings were reviewed by two pathologists (JH and RM). 24 cases with discordant p16 findings were not reclassified. From 14 cases with discordant p53 immunohistochemistry, one case was reclassified from wild type to null phenotype.

**Table 1:** Results of Cohen's Kappa Calculation Evaluating Concordance Between p16 Immunohistochemistry/qPCR and p53 Immunohistochemistry/*TP53* Mutational Analysis in NGS.

	qPCR HPV status					*TP53* mutational status (NGS)			
p16 IHC	Negative	Positive	Total		p53 IHC	Negative	Positive	Total	
Block positive	9 (12%)	68 (88%)	77	Κ = 0.62, *p* < 0.001	Mutated (aberrant)	2 (8.7%)	21 (91.3%)	23	Κ = 0.693, *p* < 0.001
Non‐block + negative	40 (73%)	15 (27%)	55		Wild type	110 (90.2%)	12 (9.8%)	122	
Total	49 (37%)	83 (63%)	132		Total	112 (77%)	33 (23%)	145	

There was a significantly worse OS (Hazard ratio = 3.33, 95% CIs 1.92–5.56, *p* < 0.0001) in p53 mutated cases (*n* = 38) compared to wild type (*n* = 171), counting overexpression and null phenotype together. Discerning p53 according to immunohistochemistry pattern, the restricted mean survival time (rmean) was roughly similar in those with p53 overexpression (*n* = 27, rmean = 2.11 years) and with null phenotype (*n* = 11, rmean = 2.53 years), but significantly worse compared to wild type (*n* = 171, rmean = 3.77 years, *p* < 0.0001, Figure 1).

## 4 Discussion

**Figure 2:**
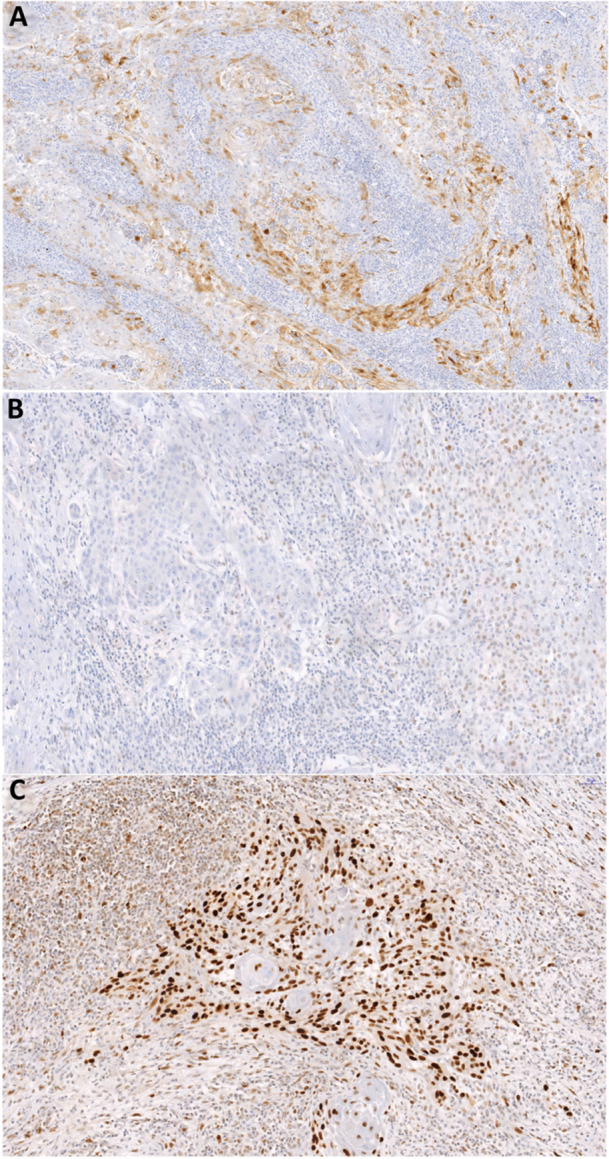
(A) penile squamous cell carcinoma with p16 positivity in ca 50% of tumor cells, evaluated as p16 non‐block positive/negative (20x), but with HPV positivity in qPCR. (B) penile squamous cell carcinoma with heterogeneous p53 expression/mutational status, null phenotype in the left part, wild type expression in the right part (20x), with *TP53* truncation mutation in NGS. (C) squamous cell carcinoma with strong nuclear positivity in > 80% of tumor cells, evaluated as mutated/overexpression (40x), but without *TP53* mutation in NGS.

PCR‐based assays detect HPV DNA and are highly sensitive, capable of identifying multiple HPV genotypes; however, they do not differentiate between transient and persistent infections. The 27.3% of cases with HPV qPCR positivity and p16 negativity could be explained by dormant inactive infection, an inactivation of the p16 encoding gene *CDKN2A* due to the loss of heterozygosity or promoter hypermethylation. In our cohort, there were two cases with qPCR HPV positivity and p16 staining in < 70% of tumor cells (Figure 2a). The 11.7% of p16‐positive but HPV qPCR‐negative cases may be false PCR‐negative due to DNA degradation and technical reasons. Alternatively, the cases may be induced by rare HPV subtypes not included in the 28 subtypes in the qPCR kit.

p53 immunohistochemistry was evaluated as wild type in 9.8% NGS positive cases, which may be caused by preanalytical factors (e.g., tissue autolysis) or tumor heterogeneity, if the tissue microdissection for NGS and immunohistochemistry were performed from separate FFPE blocks. During microscopic review, 4 of 13 cases showed heterogeneity in staining (Figure 2b). Correct interpretation of p53 immunohistochemistry is crucial. 8.7% cases with “false NGS negativity” may be explained by incorrect evaluation of immunohistochemistry slides by pathologists, as wild type expression may be very strong in > 80% tumor cell nuclei used as an arbitrary cut‐off (Figure 2c). Remaining potential explanations are the presence of a mutation located in non‐covered areas (deep intronic), DNA degradation, high non‐neoplastic cell load, and epigenetic changes in *TP53*, the typical negative regulators of which include Mdm3 or MdmX and several viruses, which may inactivate p53 by altered protein stabilization [12].

In pSCC, there have been reports describing almost perfect agreement of p16 with molecular HPV tests [13, 14, 15], similar to our results (Κ between 0.6 and 0.7) [16] but also notably lower compared to our results [17, 18]. In head and neck squamous cell carcinoma (HNSCC), the concordance between p16 and HPV detection by qPCR reached a Cohen's kappa value of Κ = 0.65 [19], similar to our results. HPV E6/E7 mRNA in situ hybridization (ISH) assay covering 18 common high‐risk types provides similar performance as qPCR in cervical, vulvar, anal, HNSCC, and their precursors [20].

In p53 immunohistochemistry/*TP53* whole exome sequencing, Trias et al. reported almost perfect agreement (Κ = 0.85) [21]. Kashofer et al. described notably lower reliability of p53 immunohistochemistry [15]. In vulvar SCC, which is etiologically, morphologically, and clinically similar to pSCC, the agreement between p53 immunohistochemistry and *TP53* NGS was similar to our results (k = 0.71, *p* < 0.001) [22]. Tessier‐Cloutier et al. described concordant results in 95% of vulvar SCCs and 93% of in situ lesions [23]. In oral squamous epithelial dysplasia, concordance of both methods was described in 92% of 57 cases [24]. The agreement between p53 immunohistochemistry and NGS on p53 has been tested across various tumor types, yielding variable results.

Mohanty et al. described the association of p53 mutated profile and nodal metastases in pSCC [25]. In line with our results, Trias et al. reported a strong detrimental prognostic impact of abnormal p53 expression in HPV‐independent pSCC [26].

We present a large cohort complex study analyzing the reliability of a technically simple and relatively cheap immunohistochemical method of p16 and p53 expression in penile cancer, yielding Cohen's kappa between 0.6 and 0.7 in both, signifying substantial agreement of immunohistochemistry with molecular testing. The strong negative prognostic impact of aberrant p53 profile in pSCC should lead to discussion about incorporating p53 testing in pathology guidelines or the WHO classification.

## Author Contributions

Jan Hrudka, Jan Hojný and Radoslav Matěj conceived and designed the study. Jan Hrudka, Jan Hojný, Eva Krkavcová, Jiří Dvořák, Zuzana Prouzová, Michaela Kendall Bártů, Nicolette Zavillová and David Čapka acquired and analyzed the data. Petr Waldauf performed the statistical analyses and prepared Kaplan–Meier survival curves. Jan Hrudka drafted the manuscript and prepared the histological figures. Radoslav Matěj supervised the study and critically revised the manuscript. All authors reviewed the manuscript, approved the final version, and agree to be accountable for all aspects of the work.

## Conflicts of Interest

The authors declare no conflicts of interest.

## Supporting information

Supporting File 1

Supporting File 2

## Data Availability

Research data are available as Supplementary material.
